# RNAi mechanisms in Huntington’s disease therapy: siRNA versus shRNA

**DOI:** 10.1186/s40035-017-0101-9

**Published:** 2017-11-27

**Authors:** Sebastian Aguiar, Bram van der Gaag, Francesco Albert Bosco Cortese

**Affiliations:** 1 Molecular Neuroscience Laboratory, Swammerdam Institute for Life Sciences (SILS-CNS), University of Amsterdam, Amsterdam, Netherlands; 2 Fulbright Program, US Department of State (IIE), New York City, NY USA; 3 Department of Neurobiology, Care Sciences and Society, Karolinska Institute, Stockholm, Sweden; 4 Biogerontology Research Foundation (BGRF), Oxford, UK; 5 Department of Biomedical and Molecular Sciences, Queen’s University School of Medicine, Queen’s University, Kingston, Canada

**Keywords:** RNAi, shRNA, siRNA, Huntington’s disease, Off-target effects, Huntingtin, Silencing

## Abstract

Huntington’s Disease (HD) is a genetically dominant trinucleotide repeat disorder resulting from CAG repeats within the Huntingtin (HTT) gene exceeding a normal range (> 36 CAGs). Symptoms of the disease manifest in middle age and include chorea, dystonia, and cognitive decline. Typical latency from diagnosis to death is 20 years. There are currently no disease-modifying therapies available to HD patients. RNAi is a potentially curative therapy for HD. A popular line of research employs siRNA or antisense oligonucleotides (ASO) to knock down mutant Huntingtin mRNA (mHTT). Unfortunately, this modality requires repeated dosing, commonly exhibit off target effects (OTEs), and exert renal and hepatic toxicity. In contrast, a single AAV-mediated short-hairpin RNA (shRNA) dose can last years with low toxicity. In addition, we highlight research indicating that shRNA elicits fewer OTEs than siRNA when tested head-to-head. Despite this promise, shRNA therapy has been held back by difficulties controlling expression (oversaturating cells with toxic levels of RNA construct). In this review, we compare RNAi modalities for HD and propose novel methods of optimizing shRNA expression and on-target fidelity.

## Background

Huntington’s Disease (HD) is a genetically dominant trinucleotide repeat disorder caused by CAG repeats within the Huntingtin (HTT) gene (chromosome 4p16.3) exceeding a normal range (> 36 CAGs). The CAG repeat is known as a polyglutamine (polyQ) tract and its length determines the severity and onset age of the phenotype. The population occurrence rate is 7 out of 100,000 in people of European ancestry. Symptoms of the disease manifest in middle age and include chorea, dystonia, cognitive decline and behavioral difficulties. Typical latency from diagnosis to death is 20 years.

The inheritance pattern of the disease is an autosomal dominant; with a 50% risk if a parent is a carrier. Age of onset and severity can be modified slightly by environment and other modifying genes.

PolyQ repeats are unstable during replication and instability increases with the number of repeats. So even if one parent has an intermediate number (28–35), the offspring may receive a higher copy leading to full penetrance. This phenomenon is known as *genetic anticipation,* where the age of onset becomes earlier in subsequent generations.

**Fig. 1:**
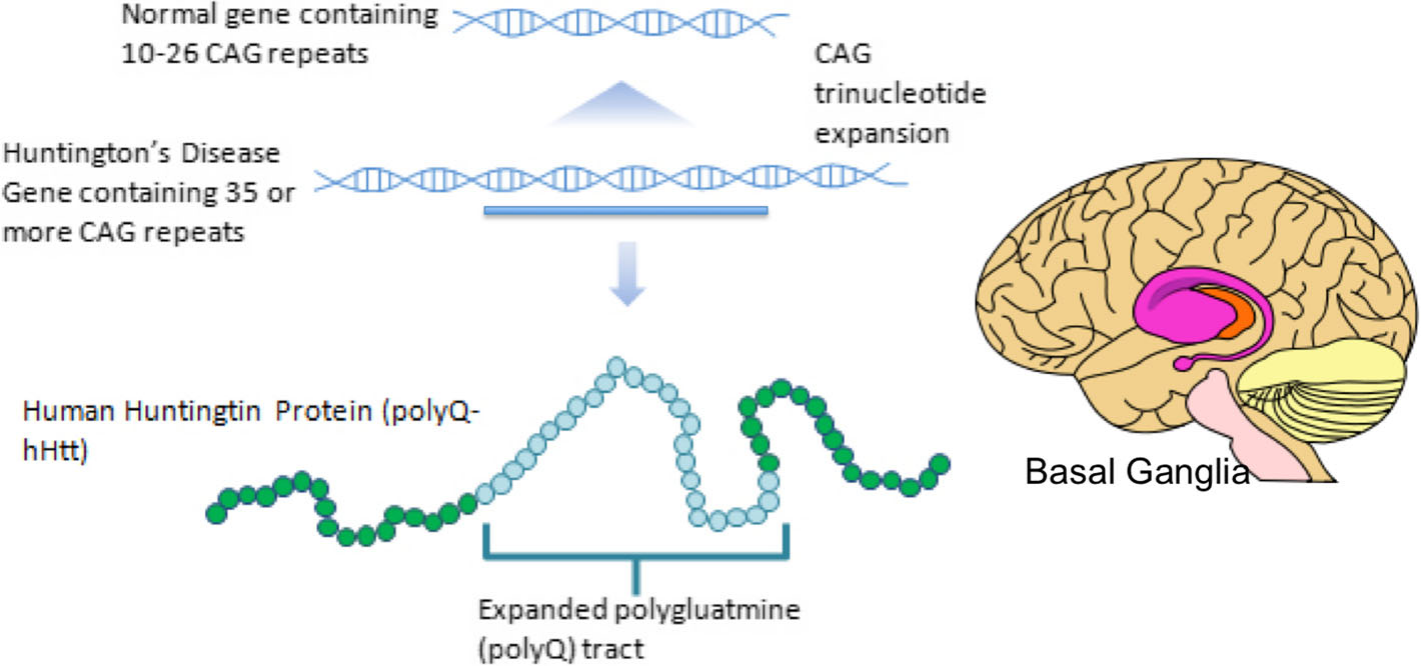
Huntingtin mutation and basal ganglia. Huntington’s results from an expanded polyglutamine tract, encoded by CAG repeats. The basal ganglia is the most severely affected region in HD

Despite accounts of disinhibited behavior increasing reproductive fitness of affected individuals, there is no evidence that they have more children. The mutant HTT gene does not confer any salutary effect, other than a possible lower incidence of cancer, perhaps because p53 is activated in HD [1, 2] Fig. 1.

### Pathology

The exact molecular pathology of Huntington is an area of ongoing research. HTT is expressed in all mammalian cells and is known to interact with over 100 genes. The highest expression levels are in brain and testes [3]. Although homozygous deletion for the HD gene is embryonic lethal in mammals, humans that are heterozygous (+/−) for the gene are normal [4].

Splicing leaves behind some polyQ peptides, which form H-bonds and aggregate. They then form inclusion bodies, an early sign of the pathology [5]. HD shows increased excitotoxicity, glial activation, and an increase in astrocytes [6], as well as altered an epigenetic pattern [7]. HTT may interfere with intercellular interactions like trophic support and can be ameliorated with ectopic BDNF [8]. Mutant HTT inhibits axonal transport of BDNF-containing vesicles in corticostriatal neurons [9].

It is not well understood why, but HTT affects certain cell types selectively — particularly neurons of the basal ganglia and especially striatal medium spiny neurons where cytotoxicity manifests earliest. Other affected areas include the substantia nigra and layers 3, 5, and 6 of the cerebral cortex [10].

### Therapeutic strategies

Many HD therapeutics target downstream consequences and symptoms of the causal pathogenic mutation in the Huntingtin (HTT) gene, while a few next-generation therapies target mHTT itself (see Table 1) [11]. The only approved drug is tetrabenzene, which palliates motor abnormalities. There are no disease-modifying therapies currently available to patients. It is known that conditional silencing of transgenic mutant HTT (mHTT) reverses HD in mice, showing that mHTT is required for HD progression [12].

**Table 1:** Disease Modifying Experimental Therapies for HTT

Strategy	Pro and Con	Citations
Humanized synthetic ZFN-KRAB repressors	+ no risk of DSBs - off target effects - triggers innate immune responses - temporary effects depending on protein turnover	Garriga-Canut, M. *et al.* (2012) Synthetic zinc finger repressors reduce mutant huntingtin expression in the brain of R6/2 mice. *Proc. Natl Acad. Sci.* [54].
CRISPR knockout of mHTT	+ permanent - too large to fit in AAV - requires PAM site near PolyQ tract - bacterial origin of Cas9 elicits innate immune response - CAG repeats within sgRNA form secondary structure, limiting efficiency	Malkki H. (2016) Selective deactivation of Huntington disease mutant allele by CRISPR–Cas9 gene editing. *Nature Reviews Neurology*.
Intrabodies	- immunogenic when injected as naked protein - Nucleic acid delivery requires a large vector such as lentivirus, which integrates genomically and can cause cancer	Cardinale, A *et al*. (2008). The potential of intracellular antibodies for therapeutic targeting of protein-misfolding diseases. Trends in Molecular Medicine [55].
siRNA/miRNA and Antisense Oligonucleotides (ASOs)	+ drug-like properties, more suited to regulation than gene therapy requiring viral vectors + can be easily customized for allele specificity + symptoms can improve for longer than the period of mRNA knockdown (“Huntingtin Holiday”) See Note 1. - short acting effect, requires long-term continuous dosing - renal and hepatic toxicity, non-trivial off target effects - inflammatory when recognized by extracellular toll-like receptors	Kordasiewicz, H. B. *et al.* Sustained therapeutic reversal of Huntington’s disease by transient repression of huntingtin synthesis. *Neuron*. Rao et al. (2009) siRNA vs. shRNA: Similarities and differences. J. Advanced Drug Delivery Reviews [56].
shRNA-based RNAi	+ longer lasting but not permanent (months to years in primates) + can fit inside an AAV, episomal plasmid in nucleus + shRNA is virally encapsulated and elicits less inflammation from toll-like receptors + constructs can be inserted into an artificial miRNA scaffold to mitigate neurotoxicity specifically - overdose due to excessively strong promoters is common - off target effects can occur	Davidson, B (2008). Artificial miRNAs mitigate shRNA-mediated toxicity in the brain: Implications for the therapeutic development of RNAi. *PNAS* [57]. See also [58, 59].

Note 1: Symptoms are reversed for longer than the period of HTT knockdown [60], known as a ‘Huntingtin Holiday,’ theoretically enabling cellular repair to occur [61]

Note 2: Designer RNAi possible based on SNPs in loci nearby to the polyQ tract, to prevent theoretical problems associated with WT HTT silencing

**Fig. 2:**
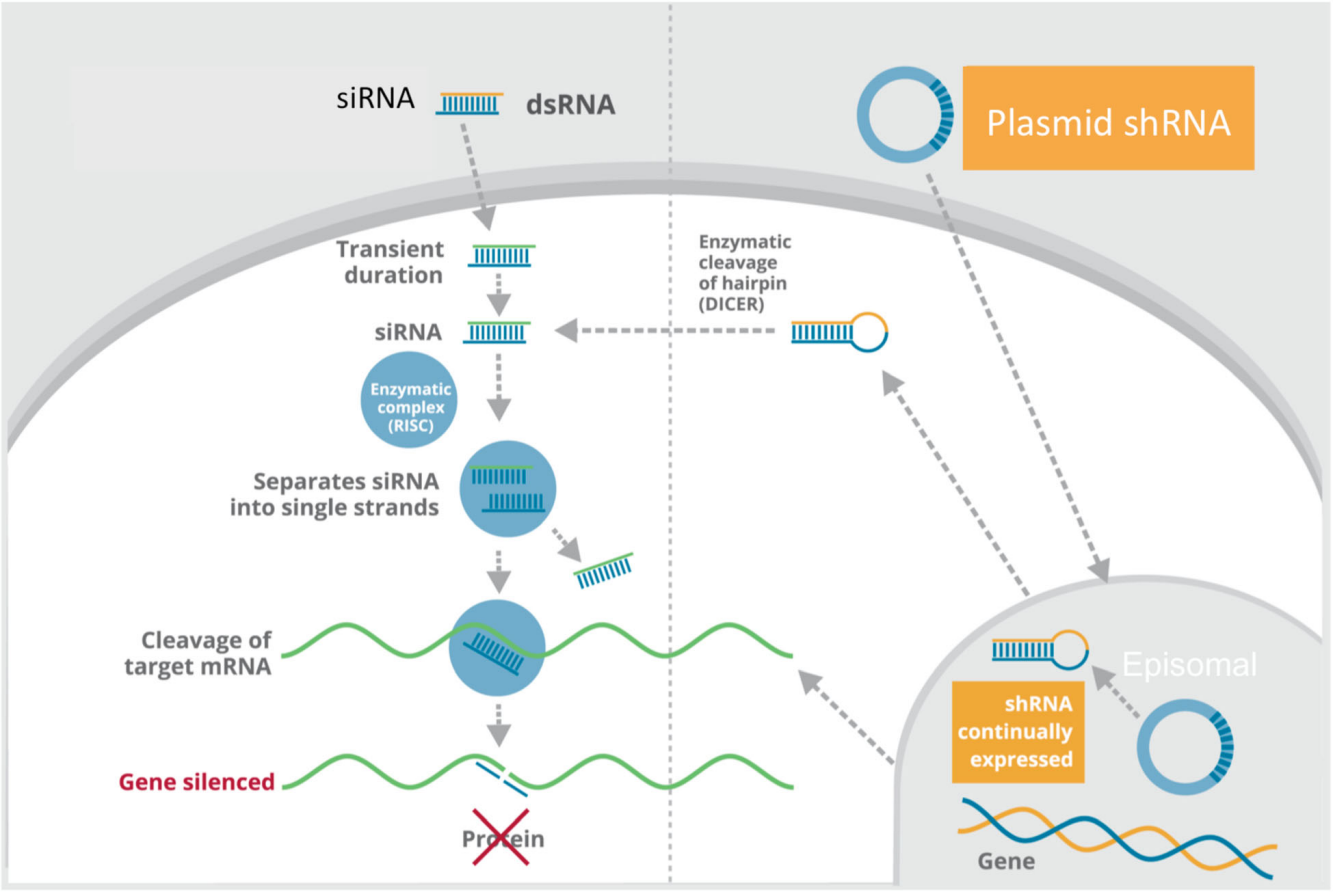
Mechanisms of siRNA versus shRNA. shRNA may be episomal or integrate into the genome via lentiviral transfection for greater stability. Both pathways converge at the RISC complex

Small molecule interventions for HD are an exciting (albeit most palliative) area of research, but beyond the scope of this review [13–20]. Targeting the primary cause of HD has become possible in recent years due to advancements in RNA interference by small noncoding RNAs (sncRNAs), such as synthetic siRNA and shRNA [21]. Despite the variations in RNAi constructs, all act by binding the mRNA of a target gene to either block translation or cause degradation of the transcript Fig. 2.

shRNA is a synthetic RNA molecule with a *s*hort *h*airpin secondary structure. Because it is delivered on a DNA plasmid rather than as double stranded RNA (e.g., siRNA), shRNA can be continually expressed for months or years.

After transcription, the product mimics pri-microRNA and is processed by Drosha to create pre-shRNA that is exported from the nucleus by Exportin 5. Then the pre-shRNA is processed by Dicer and binds to RISC complex. The passenger strand is degraded, and the anti-sense guide strand directs RISC to degrade complementary target mRNA (such as HTT).

**Fig. 3:**
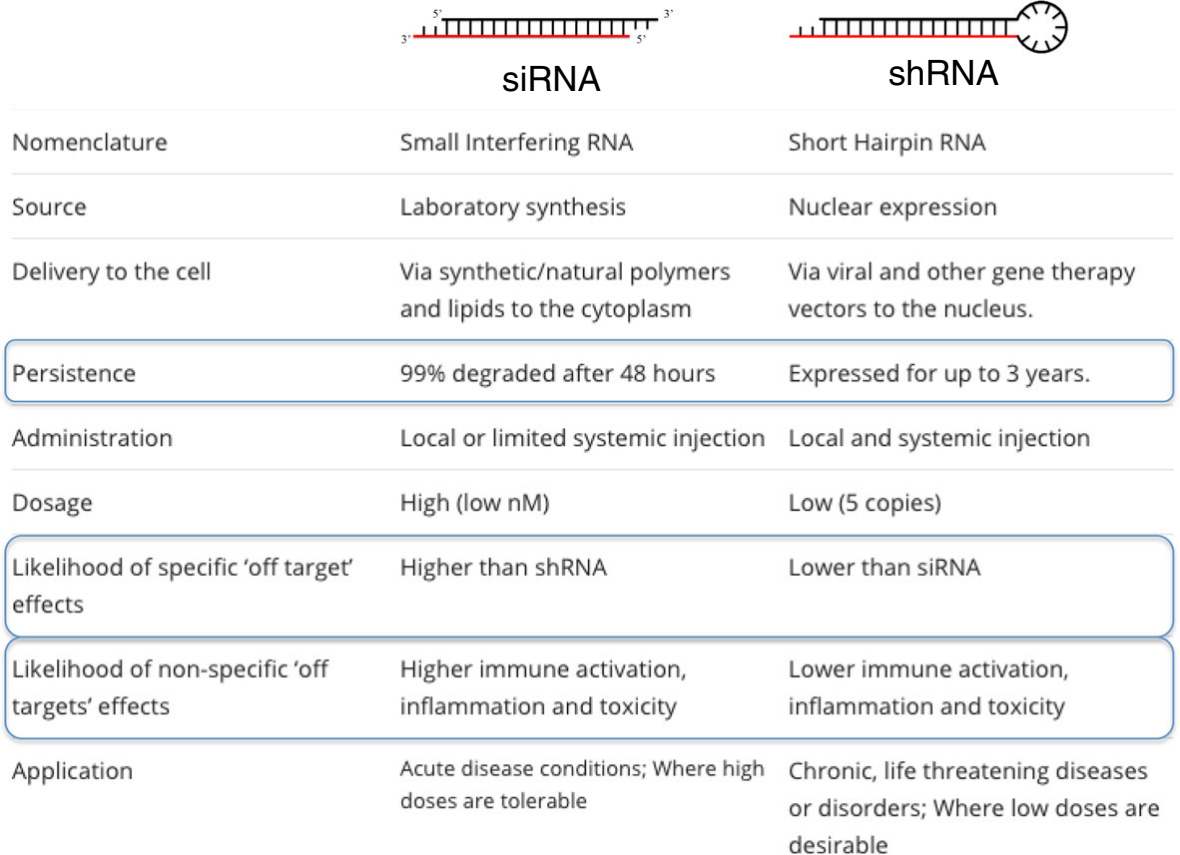
Comparison of shRNA and siRNA. Notably, shRNA does not require regular dosing that siRNA-based therapeutics do

Most HD RNAi therapies to date have been based on synthetic siRNAs or antisense oligonucleotides (ASOs) delivered naked, conjugated to cholesterol, or with lipofectamine. Unfortunately, these drugs require repeated dosing, commonly exhibit off target effects, and exert renal and hepatic toxicity [21] Fig. 3.

### Off-target effects (OTEs): shRNA vs siRNA

The off-target accuracy of shRNA versus siRNA is an open question. A few studies have indicated that siRNA has more off-target effects (OTEs) than shRNA when compared head-to-head.

Mehaffey et al. [22] treated HCT-116 carcinoma cells with either an siRNA duplex or an inducible shRNA of the same core sequence, targeting the TP53 gene, and analyzed gene expression changes 24 h post-treatment via microarray hybridization. They found a substantially higher proportion of off-target genes upregulated or downregulated in cells treated with siRNA rather than shRNA. The degree of on-target knockdown was comparable.

As a follow up to this study, Klinghoffer et al. [23] at Merck & Co repeated this experiment and included additional mRNA targets: CDKN1A, E2F1, EZH2, FDXR. The shRNA was compared to siRNA at various concentrations, and the authors also used a cell line stably expressing their constructs to control for differential transfection efficiencies. The results confirmed the prior work, showing both a greater knockdown by shRNA and fewer off-target effects (For CDKN1A, 470 transcripts downregulated by siRNA versus 19 by shRNA – of which two were shared). When lentivirus-transduced or expressed from a stable inducible cell line, shRNA showed considerably less OTEs than transfected siRNA with the same 19-mer core sequence. Titrating lower doses of siRNA could not achieve the same signal to noise ratio as shRNA. The authors used a single promoter (H1) – attempts using promoters of varying strength may yield more control over the system.

Comparing large scale RNAi screens, two using shRNA [24, 25] and the other using siRNA [26], the shRNA screens showed minimal OTEs whereas the siRNA screen found OTEs to “dominate” the results.

Another group found shRNA to be significantly more potent than siRNA on a molar basis [27]. The differential potency and accuracy of shRNA versus siRNA may be explained by a few factors: shRNA is treated more like endogenously occurring pri-miRNA hairpins – an shRNA transcript driven by a promoter such as RNA polymerase II (instead of the typically used RNA PolIII promoter) is polyadenylated, processed by Drosha in the nucleus, subject to normal nuclear export, and loaded onto the RISC complex in the cytoplasm [28].

siRNA does not undergo such processing, requires higher concentrations and frequent dosing to achieve comparable knockdown. Unprotected siRNA in the cytoplasm may be vulnerable to degradation and modifications that reduce on-target binding.

shRNA can be further optimized in the form of artificial pri-miRNA transcripts. This is achieved by by embedding the shRNA sequence into a miRNA context such as the miR-30 stem loop precursor. This method was used to inducibly control p35 levels in vitro and in vivo, even when the construct was present as a single genomic copy [29].

Based on this artificial pri-miRNA strategy, the Dutch gene therapy company UniQure has achieved mutant HTT allele-specific targeting via AAV5 in a humanized HD mouse model [30].

### Tough decoy RNAs (TuDs)

Regardless of the actual degree of off-targeting events in shRNA, complementary strategies to minimize (already relatively low) shRNA OTEs are emerging. One such strategy to deter off-target gene perturbation is the use of “tough decoy RNAs” (TuDs), which are competitive decoy RNAs with high complementarity for the shRNA sense strand. Such TuDs are designed to bind to the shRNA sense strands before they have an opportunity to associate with non-targeted genes.

Mockenhaupt et al. [31] used TuDs co-expressed with shRNAs designed to target AAV-delivered hepatitis C virus to simultaneously reduce shRNA off-targeting and potentiate off-target inhibition, managing to reduce the number of off-targeted genes from 334 without TuD coexpression to 186 genes with TuD coexpression. Notably, their approach can be applied to existing shRNA constructs, in contrast to all previously-reported strategies, which require the de novo construction of shRNAs optimized for sense-strand specificity.

### Further shRNA considerations

shRNA offers longer-lasting treatment, but delivery has been a challenge until recently. shRNA can be delivered in vivo using a viral vector. Adenoviral-associated viruses (AAVs) are the vectors of choice in clinical trials because of their low immunogenicity, numerous engineered tissue-specific serotypes, and very low rate of chromosomal integration (preventing insertional mutagenesis) [32].

The key to the safety of AAVs is that the construct they deliver remains episomal. In the last 2 years, two AAV-based gene therapeutics have been approved (GlaxoSmithKline’s SCID therapy and uniQure’s lipoprotein lipase deficiency therapy), with many more AAV-based gene therapies currently in clinical trials [33].

HD researchers have also neglected shRNA because early studies showed overdoses, where the non-coding RNA machinery was overpowered by the exogenous shRNA and endogenous ncRNAs could not compete [34]. Other studies since have shown dramatic, longer-term improvement in various diseases using shRNA [35].

siRNA has been shown to improve HD pathology in vivo [36]*.* shRNA offers longer-lasting knockdown and reduced hepatic and renal toxicity. The main challenge facing shRNA is intracellular overdose – clogging up the miRNA processing machinery. Altering the strength of shRNA construct expression may offer a solution to this problem.

### Modulating promoter strength

**Fig. 4:**
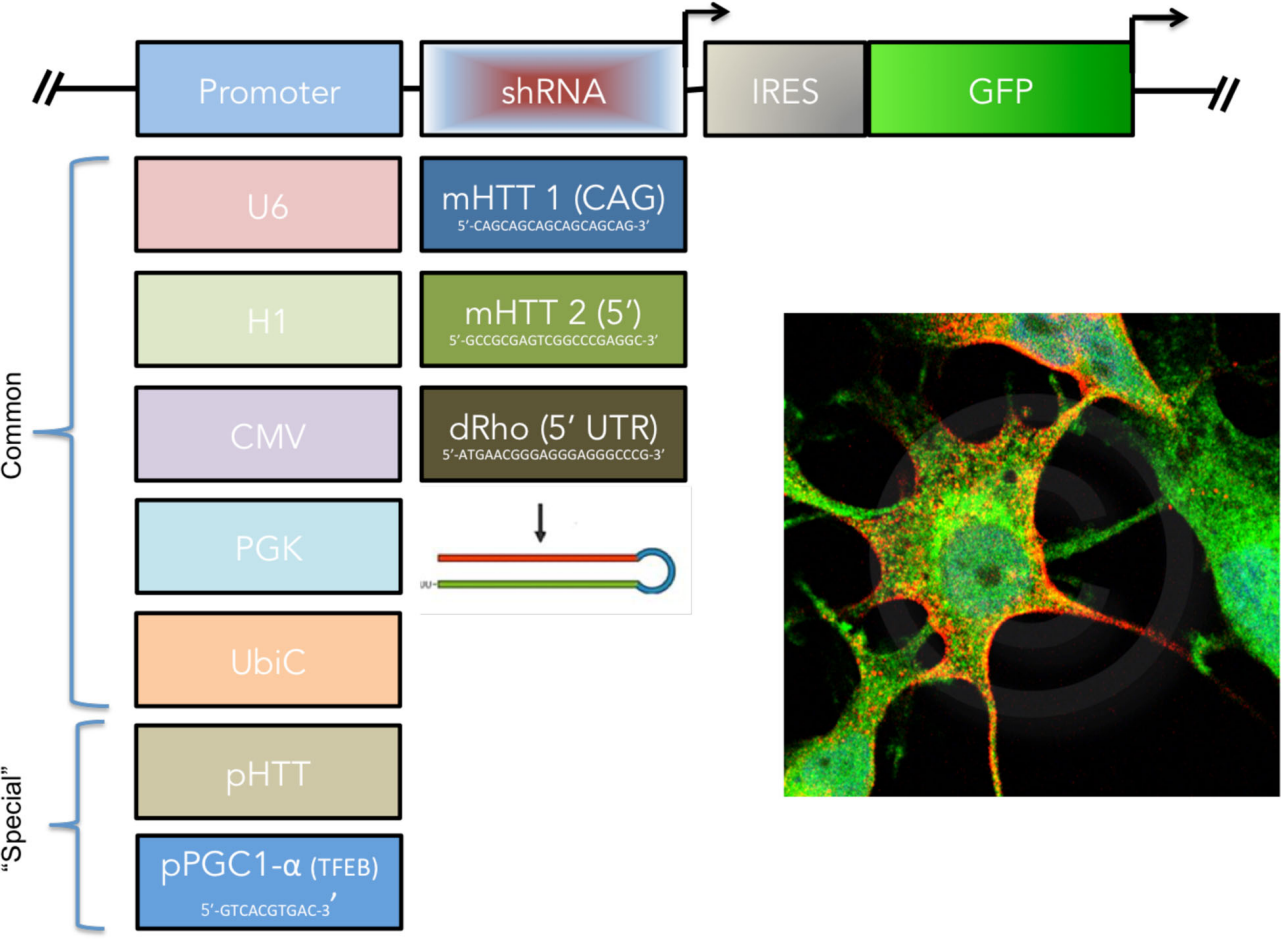
Construct design for promoter modulation. Various promoters can be coupled with mHTT variants for alle-specific silencing (and compared to a control such as dRho). An IRES followed by GFP may be used to confirm construct expression. The “special” promoters include the HTT promoter itself, and the PGC1-a promoter which is activated by TFAM when HTT aggregates induce autophagy or lysosome formation

Promoter strength is a crucial factor for shRNA knockdown efficiency and toxicity to transfected cells. Class III polymerase promoters such as U6 are commonly used in siRNA applications because they are strong promoters, enabling powerful knockdown Fig. 4.

However, excessive shRNA expression from class III promoters can result in cytotoxicity, innate immune response activation, and can even be fatal in vivo after only 1 month of sustained expression.

One landmark study by Grimm et al. [34] demonstrated that an shRNA targeted to the liver caused hepatic cytotoxicity due to high level, sustained expression in mice (the empty AAV vectors were non-toxic at doses used). Morbidity depended upon the sequence of the shRNA and was associated with downregulation of endogenous miRNAs, indicating competition with the exogenous shRNA for access to a finite supply of RNA machinery involved in RNA processing such as the nuclear karyopherin exportin-5, which is easily saturated (and toxicity was reduced after exportin-5 overexpression).

The authors were able to resolve this overdose phenomenon by optimizing the viral vector shRNA dose and by weakening the promoter sequence, successfully applying this method to the amelioration of a hepatitis B mouse model in vivo.

Multiple shRNAs could be cloned into available vectors containing various promoters, such as: U6, H1, CMV, PGK, and UbiC. These promoters vary in their intrinsic strength due to differences in CpGs promixal to the TATA box, which determine affinity to RNA polymerase. In addition, promoter strength varies as a function of cell type specific transcriptional programs [37].

### Negative feedback-driven oscillating promoters

**Fig. 5:**
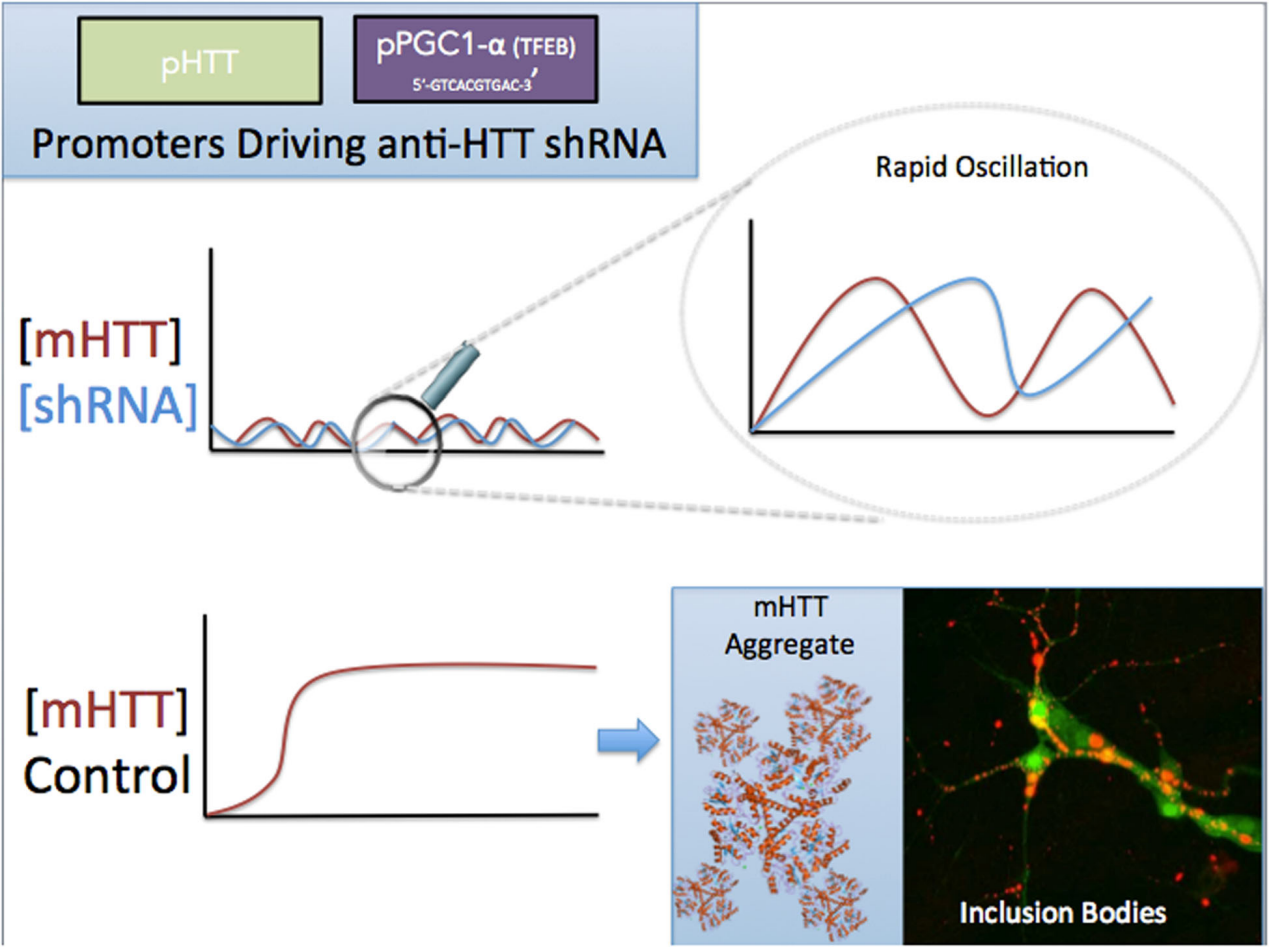
Negative feedback loop mechanism. If the promoter of HTT is used to drive the shRNA construct, a stoichiometric equivalent of shRNA may be expressed to neutralize mHTT levels. A similar strategy may be employed for constructs activated during autophagy and lysosomal biogenesis, such as PGC1a. Then, the shRNA will be expressed when HTT aggregates have formed to induce autophagy or clearance – shRNA on demand and only when required by the cell

Figure 5 given that shRNA against mHTT should be a *stoichiometric equivalent*, the human HTT promoter could be used to drive expression of the shRNA constructs. Whenever the cell expresses HTT endogenously, the shRNA construct will also be expressed at a similar frequency. This may result an oscillatory expression pattern that keeps HTT levels relatively low.

TFEB is a transcription factor and master regulator of lysosomal biogenesis and autophagy. TFEB is expressed during aberrant lysosomal conditions like in lysosomal storage diseases (LSDs), amyloid diseases (Alzheimer’s, Lewy Body dementia), and Huntington’s disease. Overexpression of TFEB reduces the pathogenic effects of mHTT in vitro [38]. TFEB is activated by PGC1a, and a TET-on conditional induction of PGC-1a was shown to ameliorate HD pathology in mice by eliminating mHTT protein aggregates and oxidative stress via TFEB [39]. Additionally, the CLEAR-box sequence (5′-GTCACGTGAC-3′ present in the regulatory region of lysosomal genes) in the PGC-1a promoter (a known binding site for TFEB) could be used to drive expression of the experimental shRNA.

**Fig. 6:**
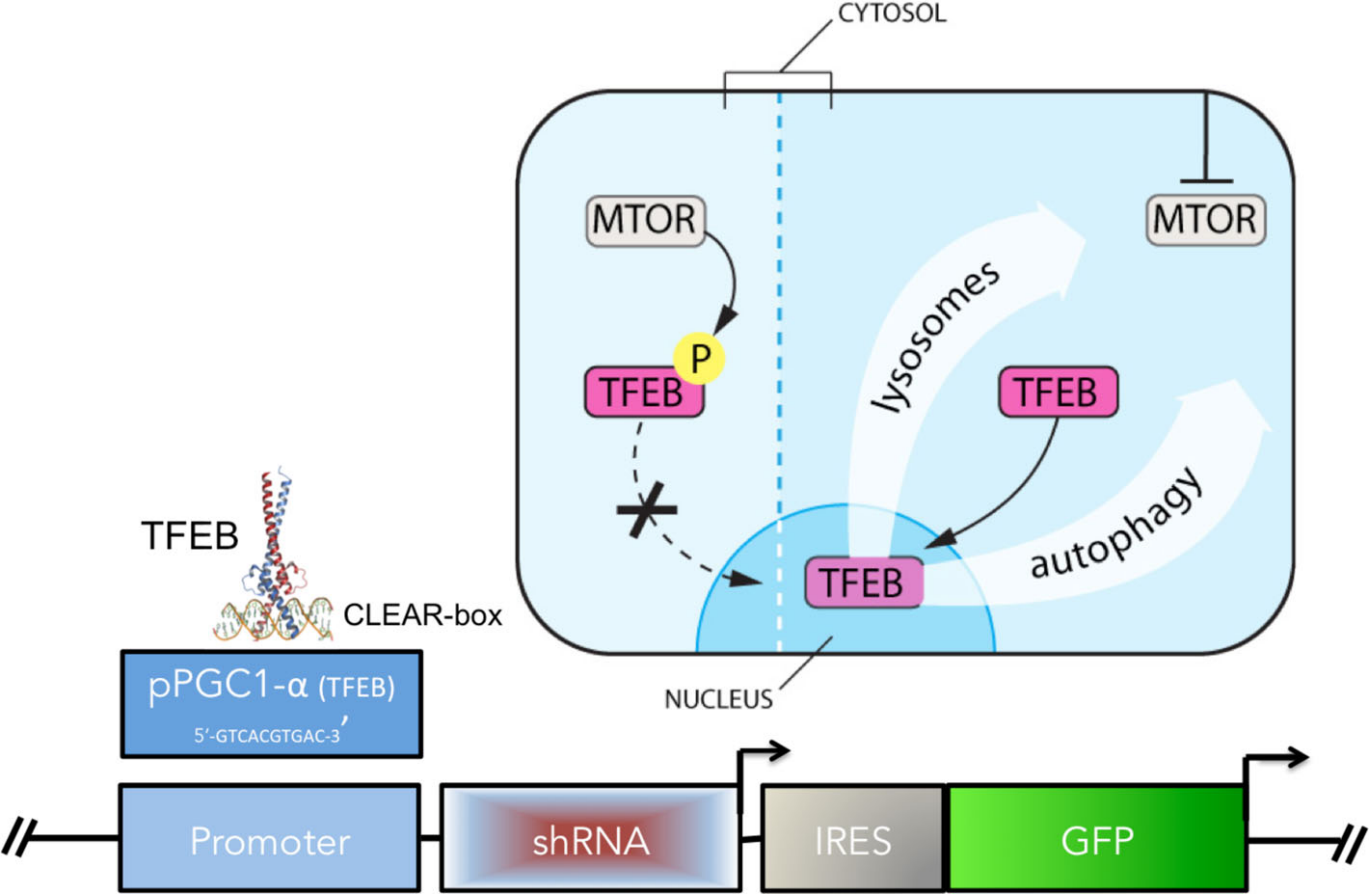
Mechanism of TFEB at the PGC1-a promoter. The PGC1a promoter contains a CLEAR-box that is known to be bound by TFEB, a transcription factor induced during autophagy and lysosomal biogenesis. A construct being the PGC1a promoter CLEAR-box would be induced by TFEB under conditions of intracellular proteotoxicity due to HTT aggregation. By this mechanism, on-demand suppression of HTT could be achieved

Cells with mHTT inclusion bodies have increased autophagy and TFEB activity. In theory, TFEB will be highly expressed only when mHTT aggregates sufficiently and induces compensatory autophagy. Autophagy and shRNA knockdown of mHTT will then both occur, potentially reducing the pathogenic burden of mHTT followed by a reduction in TFEB expression, autophagy and shRNA construct expression. This oscillation mechanism may act as a negative feedback loop to ensure that shRNA is not expressed to a deleterious degree, and only when necessary Fig. 6.

### Targeting the CNS

The Blood-Brain Barrier (BBB) is a significant challenge to CNS drug delivery. Tight junctions between endothelial cells that line the capillaries in the brain form a highly protective barrier, allowing only transport across the barrier to either small lipid-soluble molecules or to larger molecules that rely on carrier-mediated or receptor-mediated transport [40]. This is problematic for developing RNAi treatment strategies, considering that therapeutic siRNA and shRNA molecules typically are too large to allow BBB penetration. Conventional gene therapy treatment therefore often made use of direct stereotaxic surgical intervention which enabled injection of viral DNA constructs directly at the site of interest [41]. This technique is highly invasive however and leads only to localized action of treatment at the site of injection, which sometimes might be desirable, but usually the goal of most treatment strategies is to affect the whole brain. Recent advances however in viral and non-viral vector technologies have opened up new possibilities for gene therapy in the CNS through the vasculature, including RNAi. shRNA and siRNA that may be delivered to the CNS in the following ways: viral vectors (mainly the adeno-associated virus, AAV) and exosomes/liposomes.

With respect to viral gene therapy, immunogenicity of AAVs (unlike e.g., lentiviruses) has been minimal in practice [42]. AAVs are non-integrating (the construct remains episomal), reducing the chromosomal integration cancer risk that marred two early clinical trials of retrovirus-based gene therapies. Optimizing the strength of construct expression is another roadblock. AAV transduction efficiency, a key barrier to therapy, is increasing rapidly thanks to directed capsid evolution, pioneered by companies such as 4D Molecular Therapeutics [43] (indeed, in the last few years the EMA has approved three gene therapeutics -- the first was UniQure’s lipoprotein lipase Glybera in 2012, which was a commercial failure but clinical success) [44].

UniQure has achieved preclinical proof-of-concept with their Huntington’s gene therapy (AMT-130), which consists of an AAV (serotype 5) vector carrying an artificial micro-RNA. The group injected the AAV5-miRNA bilaterally into humanized MTT mouse striatum, achieving significant knockdown and attenuation of pathology [45]. UniQure and investigators at UCSF demonstrated that parenchymal administration of AAV5 (1 × 10^13^ vector genomes per milliliter) in non-human primates resulted in extensive expression throughout the brain, highlighting anterograde transfection efficiency [46]. In both cases, administration of the AAV required invasive administration into subcortical structures of the brain. Recent advances in AAVs have shown the promise of systemic and intrathecal administration routes.

Liposomes, exosomes, and comparable nanoparticles offer potential advantages over viral vectors. These nanoparticles tend to be less immunogenic than viruses, and they are amenable to modification and customization (such as PEGylation, conjugation with cell-penetrating peptides, and homing peptides for tissue tropism). The future of gene therapy may indeed feature these more customizeable nanoparticles. However, their transduction efficiency still lags behind viral vectors. Kumar et al.*,* [47] demonstrated transvascular delivery of siRNA to the brain by coupling siRNA to a rabies virus glycoprotein (RVG), which offered in vivo protection to fatal viral encephalitis in mice and mainly targeted neurons through specific binding to the acetylcholine receptor. A recent study that made use of this technique demonstrated high-efficiency (62% protein reduction) knockdown of Alzheimer’s target BACE1 through systemic injection of targeted exosomes bearing a RVG [48].

Another treatment strategy to deliver desired agents across the BBB might be through ‘loosening’ of the tight junctions, which could be achieved by targeting tight junction claudin-5 expression with siRNA intervention, which increased BBB permeability [49].

Systemic administration of CNS-targeting vectors has been demonstrated, but efficiency remains a concern. In addition, non-target peripheral tissues (especially the liver) are exposed to the drug, presenting potential toxicity risks and requiring larger quantities of the drug, which is expensive to manufacture. Systemic vector delivery to the CNS is further reviewed by Boudenx et al. [50].

Intrathecal administration into the CSF limits off-target risks due to limited exposure of peripheral tissue to the drug, and requires a lower dose of drug. Bey et al. [51] demonstrated that intrathecal AAV9 delivery either via lumbar or intracisternal injection result in robust expression of a construct bearing the neuron-specific synapsin 1 promoter driving GFP. This work compliments the results of Hordeaux et al. 2017, attempting intrathecal administration of AAV9/10 bearing acid-α-glucosidase (GAA), the aberrant gene in Pompe disease. A dramatic remediation of Pompe symptoms was achieved in a GAA-KO mouse model of Pompe disease [52]. Progress in AAV-based gene therapy for numerous CNS conditions is further reviewed by Hocquemiller et al. [53]. Delivery of AAVs and non-viral vectors to the CNS, a problem long considered a major barrier to progress, is now beginning to yield to innovative new methods.

## Conclusion

There are currently no disease-modifying treatments for Huntington’s disease. RNAi is an exciting area of therapeutic development that offers substantial patient benefit and even a cure. Recently, most research in HD RNAi has focused on synthetic siRNA and antisense oligonucleotides. Despite their advantages, these molecules have several drawbacks: frequent, high dosing results in toxicity and the drugs are not targeted specifically to the brain. In contrast, shRNA delivered by AAV can be a small single dose treatment offering long-lasting expression.

Gene therapy itself was slowed dramatically after the tragic deaths of several patients in clinical trials. In recent years, gene therapy has advanced dramatically due to innovations in the efficiency of non-integrating viral vectors (e.g., AAVs). Similarly, shRNA was held back by limitations in control over shRNA dosage per cell. By using promoters of different strength, it may be possible to finely tune and optimize shRNA expression to minimize toxicity while maximizing target knockdown. This line of research is relevant to many diseases amenable to RNAi therapy in which aberrant gene expression is a causal factor.

## Abbreviations

ASO: antisense oligonucleotides
HD: Huntington’s Disease
HTT: Huntingtin
OTE: Off target effects
shRNA: Short hairpin RNA
siRNA: small interfering RNA
TuD RNA: Tough Decoy RNA
